# High prevalence of the Mediterranean spotted fever agent *Rickettsia aeschlimannii* in *Hyalomma marginatum* ticks from Pianosa island, Italy

**DOI:** 10.1186/s13071-026-07281-9

**Published:** 2026-02-03

**Authors:** Patricia Alba, Valentina Tagliapietra, Alessia Franco, Leonardo Forbicioni, Sara Coppola, Margherita Capitani, Fiorentino Stravino, Roberta Onorati, Angela Ianzano, Daniele Arnoldi, Claudio De Liberato, Antonio Battisti, Annapaola Rizzoli

**Affiliations:** 1 Istituto Zooprofilattico Sperimentale del Lazio e Della Toscana “M. Aleandri”, Rome, Italy; 2 Fondazione Edmund Mach, Research and Innovation Center, San Michele All’Adige, TN Italy; 3 World Biodiversity Association Onlus, Sezione Arcipelago Toscano, Portoferraio, LI Italy

**Keywords:** *Rickettsia aeschlimannii*, *Hyalomma marginatum*, Pianosa island, Metabarcoding, Genomics

## Abstract

Tick-borne rickettsioses, caused by bacteria of the spotted fever group (SFG) *Rickettsia*, are emerging infectious diseases whose emergence and spread are often affected by climate and land-use changes. Here, we report the results of monitoring *Hyalomma* spp. ticks in the protected area of Pianosa island (Tuscany (Italy) and subsequently investigating their bacterial community through metabarcoding sequencing. In this study, based on analyses of 575 tick specimens distributed in 120 pools, we recorded a high prevalence of *Rickettsia aeschlimannii* (58% and 75%* gltA*- and 17 kDa-Ag protein-positive pools, respectively), an agent of the SFG group in *H. marginatum*. These findings support the need for warnings on the risk of transmission of spotted fever rickettsiosis to both residents and visitors and underline the need for enhanced surveillance, public awareness and preventive measures.

**Graphical Abstract:**

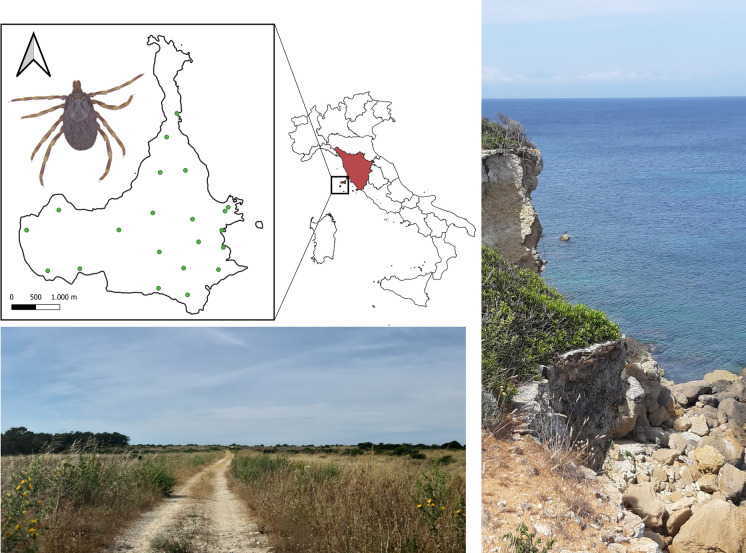

Tick-borne rickettsioses are caused by obligate intracellular bacteria belonging to the spotted fever group (SFG) of the genus *Rickettsia* [1]. The distribution of SFG rickettsioses varies geographically and correlates with the distribution of their tick vectors. In Italy and Europe, Mediterranean spotted fever (MSF) has found to be associated with different *Rickettsia* species in addition to *R. conorii*, including *R. monacensis*, *R. massiliae*, *R. slovaca*, *R. helvetica*, *R. sibirica, R. raoulti, R. rioja* and *R. aeschlimannii* [2, 3]. Land use, livestock farming and climate change are considered to be important drivers of rickettsiosis emergence, altering not only habitats but also tick distribution and behaviour, including those of their hosts [4]. In the framework of an eco-epidemiological monitoring programme focusing on *Hyalomma* spp. ticks carried out in the protected area of Pianosa island (Tuscany, Italy), we investigated the bacterial microbiota of these ticks through metabarcoding sequencing.

Pianosa island (10°04′44″E, 42°35′07″N) is the fifth, by extension, of the seven islands of the Tuscan Archipelago National Park in Italy (www.islepark.it), with a total area of 10.25 km^2^. Since historical times, the island has undergone intensive changes in land use and animal farming. From 1856 until 1998, it served as a penal colony, characterized by intense farming and breeding activities involving various introduced livestock species, such as pigs, cows, sheep and chickens. It became a natural protected area in 1996 (Parco Nazionale dell’Arcipelago Toscano; www.islepark.it), resulting in most of the farming and agricultural activities being suspended. In 2014, a LIFE Nature project [5] was launched for habitat restoration, which included the eradication of non-indigenous vertebrate species such as livestock, rodent pests, domestic cats, hedgehogs and game birds. This project also clarified the genetic status of the local population of European brown hare,* Lepus europaeus* subsp. *meridiei* (Hilzheimer, 1906). This isolated population is considered to be a relic and unique endemic stock of the autochthonous European brown hare (*L. europaeus* Pallas, 1778) due to its isolation and distinct genetic characteristics [6]. Constrained by the island’s limited area (10 km^2^), the local hare population exhibits a high population density (about 260 hares; 0.26 hare/ha) [7], providing an abundant and easy accessible source of blood for ticks. Pianosa island is one of the few important refuge locations where this lineage still persists [8]. The island is also part of the Natura 2000 Network, designated as both “Special Protection Area (IT5160016)” and “Site of Community Importance (IT5160013)”, due to its significant ornithological value as a stopover site for migratory birds (e.g. *Streptopelia turtur*, *Ciconia ciconia*, *Apus pallidus*, *Phoenicurus phoenicurus*), and as a breeding site for several bird species of community interest, such as *Lanius minor*, *Caprimulgus europaeus*, *Calandrella brachydactyla*, *Calonectris diomedea* and *Ichthyaetus audouinii*. Migratory birds are recognized as important hosts for ticks, including *Hyalomma* spp., and tick-borne pathogens, as has also been previously reported on other small Italian islands [9–11]. Currently, the island is a prominent tourist destination, host to 15,000–20,000 visitors annually [12]. However, to preserve its fragile ecosystem, the daily number of visitors is restricted to a maximum of 250 people.

We collected *Hyalomma* spp. ticks from the environment using CO_2_ traps (Fig. 1b) activated with dry ice for 24 h during 11 trapping sessions, from August 2021 (preliminary survey) to February 2023 (Table 1). In total, 21 sampling points, each equipped with one CO_2_ trap, were randomly distributed across the island (Fig. 1a). The number of ticks collected per trap varied greatly, ranging from zero to 232, with a peak, in the number of total specimens caught, in August (Table 1). From each trap and session, we collected a subsample of 30 ticks in vials and sent these alive to the Department of General Diagnostics, Istituto Zooprofilattico Sperimentale del Lazio e della Toscana (IZSLT; Veterinary Public Health and Food Safety Institute, Rome, Italy). Among these, 10 ticks were randomly taken and placed in two vials of five individuals each. The numbers of subsamples and pooled ticks varied depending on sample availability and the conditions per trap and session. Occasionally, dead wild animals were also collected and brought to the same laboratory for further investigation. Thirty samples from different organs (liver, spleen, lung, brain, kidney, heart, gut content, skin, nasal and tracheal swabs) were obtained from four wild animal carcasses (two brown hares, *Lepus europaeus* subsp *meridiei*; one yellow-legged gull, *Larus michahellis;* and one house mouse, *Mus musculus*) and subsequently processed for extraction (Table 1).

**Fig. 1:**
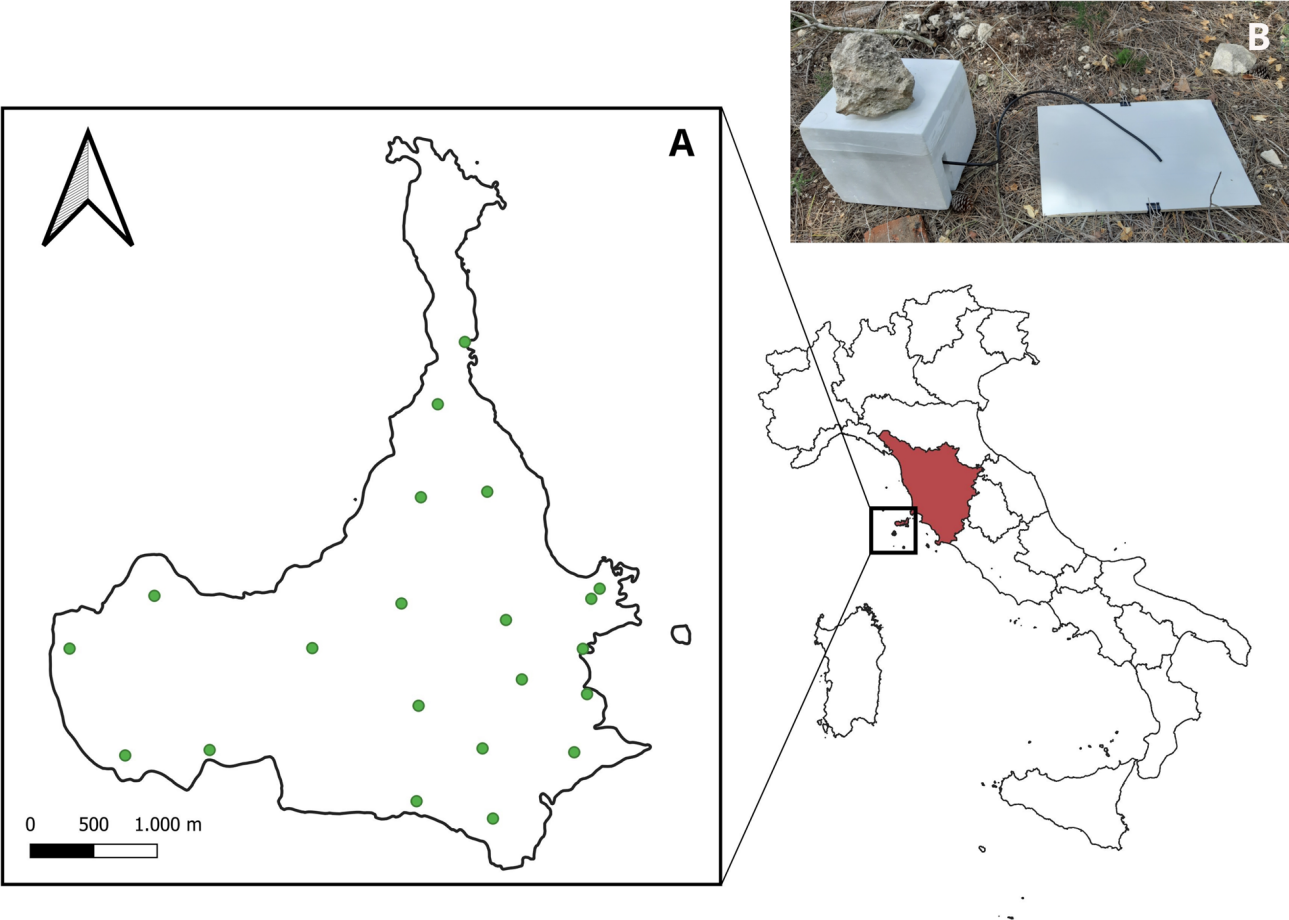
**A** Map of Pianosa island (Archipelago Toscano National Park, Italy) and monitoring sites (green circles). **B** Example of CO_2_ traps used for the collection of *Hyalomma* ticks

**Table 1:** List of organs per species of wild dead animals, analysed for the presence of *Rickettsiales*, Pianosa island, Italy, 2021–2023

Species/type of samples	*Lepus europaeus* subsp *meridiei* (animal 1)	*Lepus europaeus* subsp *meridiei* (animal 2)	*Larus michahellis*	*Mus musculus*
Liver	X	X	X	X
Spleen	X	X	X	X
Lung	X	X	NA	X
Brain	X	X	X	X
Kidney	X	X	NA	X
Heart	X	NA	X	NA
Small intestine content	X	X	NA	NA
Caecal content	X	NA	X	X
Skin	X	NA	NA	NA
Nasal swab	X	X	NA	NA
Tracheal swab	X	X	NA	NA

*NA* Sample not available,* X* sample collected

Total DNA from the samples was extracted using the QIAsymphony Path/virus kit (Qiagen, Hilden, Germany) after mechanical homogenization and a 60-min incubation (overnight for the animal tissue samples) with proteinase K and lysozyme; swabs were diluted directly in the extraction kit buffer according to manufacturer's instructions. The purified DNA was amplified with universal 16S ribosomal RNA (rRNA) gene primers for the V3-V4 regions, with Illumina overhang adapters (Illumina Inc., San Diego, CA, USA), using Platinum II Taq Hot-Start DNA Polymerase in a ready master mix. PCR cycling conditions consisted of an initial denaturation at 95 °C for 3 min; followed by 35 cycles of denaturation at 95 °C for 15 s, annealing at 54 °C for 15 s and extension at 72 °C for 5 s; with a final extension at 72 °C for 3 min and a hold at 4 °C. Amplicons were purified and attached to the Illumina indexes (Nextera XT Index Primers) following the manufacturer’s protocol and then sequenced using Illumina technology (MiSeq). Raw reads were analysed using the Qiime2 suite-tool [13]; in particular, the Dada2 pipeline [14] was used for filtering and cleaning. Taxonomical classification was performed using the Silva-138 database [15] after a clusterization step of the obtained amplicon sequence variants (ASVs) with a 99% similarity.

*Rickettsiales* species identification was carried out using amplicon sequencing, following the aforementioned wet-lab protocol but modifying the first PCR annealing temperature (Ta) for two specific genes: the citrate synthase-encoding gene (*gltA; *Ta = 58 °C) and the 17-kDa antigenic protein-encoding gene (17 kDa-Ag protein; Ta = 54 °C) of *R. rickettsia*, as previously published [16]. The most prevalent AVSs were identified using the Basic Local Alignment Search Tool (BLAST) with the nr/nt (non-redundant nucleotide) database.

The presence of *H. marginatum* ticks on Pianosa island (Italy) has been reported since the late 1990s and are considered to be a nuisance by residents and tourists, especially during the summer months. In this study, we were able to assess the widespread occurrence of this tick species by collecting samples from the ground, and to evaluate the presence and prevalence of *R. aeschlimannii*, considered to be the main agent of the Mediterranean SFG, through metabarcoding in both ticks and in wildlife carcasses that were occasionally found.

In total, 1685 tick samples were collected using CO_2_ traps and morphologically identified [17], among which 1683 tick samples were identified as *H. marginatum* (1660 adults and 23 nymphs) and two were identified as *Ixodes ventalloi* (adults). Among the 1032 adult ticks sent to the laboratory, 575 were selected for further analysis; these were randomly placed in 120 pools and subsequently extracted (Table 2). The 16S-targeted analysis indicated that > 30% of the samples contained the following bacterial genera in their microbiota: *Mycobacterium* spp., *Williamsia* spp. *Rickettsia* spp., Candidatus *Midichloria*, *Francisella* spp. and *Pseudomonas* spp. Analysis for *Rickettsiales* in the *H. marginatum* pools revealed that 70/120 pools were positive for *gltA* (58.34%, 95% confidence interval [CI] 48.98–67.26%) and 90/120 pools were positive for the 17 kDa-Ag protein (75.0%, 95% CI 66.27–82.45%). Animal tissue samples were 100% positive for both target genes. All amplicons (from positive pools and tissue samples) were identified as *R. aeschlimannii* [2, 18].

**Table 2:** Number of *Hyalomma marginatum* ticks per year and month, including specimens sent to the laboratory and number of pools analysed (Pianosa island, Italy, 2021–2023)

Year	Month	Number of ticks	Number of specimens sent to laboratory	Number of tick pools
2021^a^	August	627	457	20
2022	June	332	124	10
2022	August	573	303	42
2022	September	94	91	23
2022	October	27	27	14
2022	November	13	13	0
2023	January	9	7	8
2023	February	10	10	3
Total		1685	1032	120

^a^Preliminary field survey data

*Hyalomma marginatum* is a known vector of significant emerging infectious diseases such as Crimean-Congo haemorrhagic fever (CCHF) and MSF. To complete its life-cycle, immature stages feed mainly on birds and small vertebrates (Rodentia and Lagomorpha), while adults mostly prefer large ruminants of the Bovidae family [19]. Notably, the life-cycle and persistence of *H. marginatum* on Pianosa island seems to be sustained by the local population of *L. europaeus* subsp. *meridiei*, on which all tick stages were detected (Fig. 2).

**Fig. 2:**
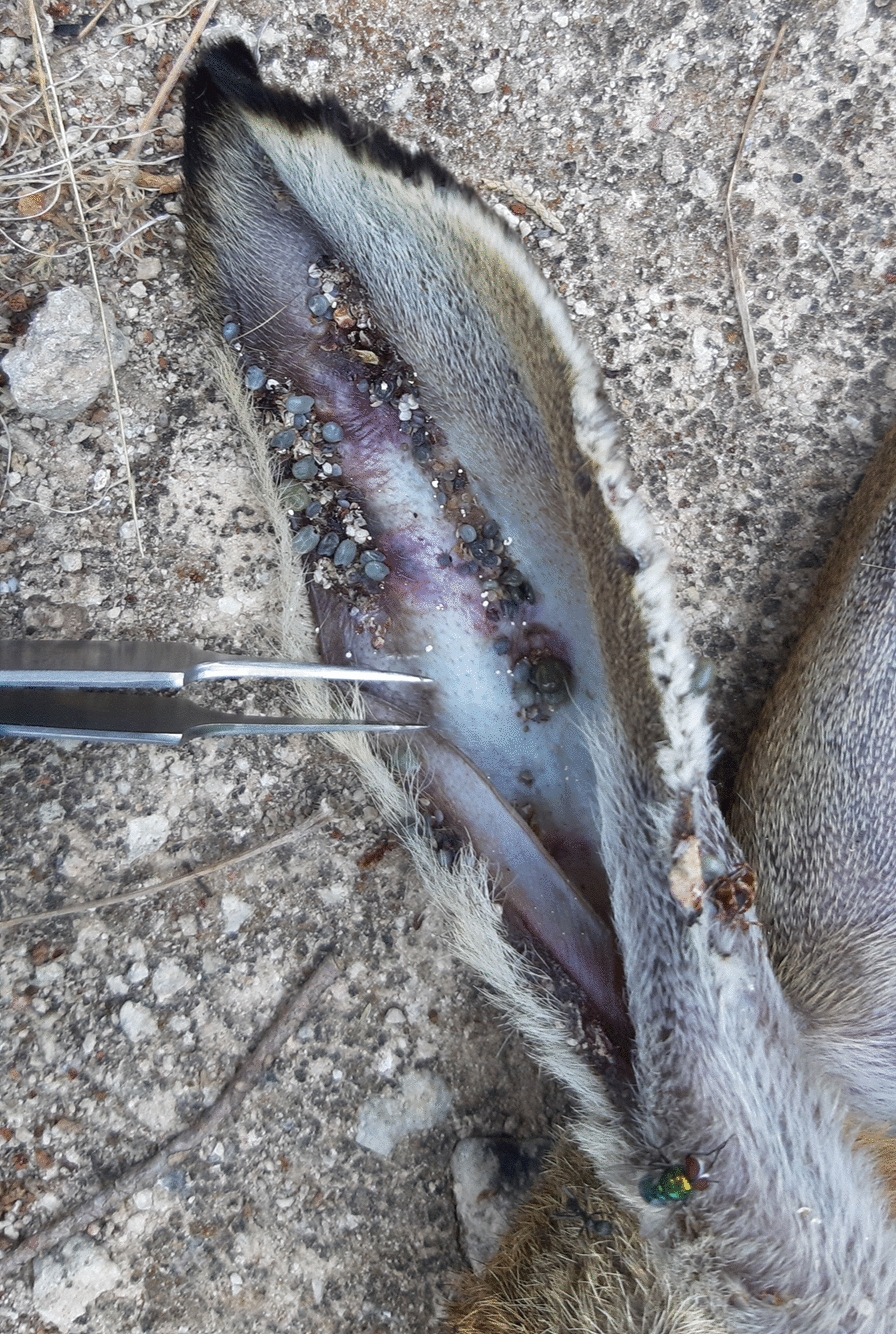
*Hyalomma* spp. ticks on the ear of a dead European brown hare, *Lepus europaeus* subsp. *meridiei* (Pianosa Island, Italy). Photograph provided by V. Tagliapietra

*Rickettsia aeschlimannii* is widespread across Europe, and its transmission is further facilitated by tick dispersal via migratory birds carrying infected ticks [20–23]. While its occurrence in ticks belonging to the genus *Hyalomma* has been already reported in Italy [20, 24] and other European countries [25], our study is the first to report such a high prevalence in host-seeking specimens collected from the field. A recent study in the Mediterranean area (Occitanie region, France) [26] also revealed a high prevalence of *R. aeschlimanni* in *Hyalomma* ticks, including egg pools from infected females. The authors of the French study considered their results to be suggestive of a possible role as a tick symbiont in *H. marginatum*. However, the 100% positivity of the samples in the dead animal tissues/organs examined in our study also suggests a potential sustained transmission cycle of the pathogen between ticks and hosts.

MSF cases due to *R. aeschlimannii* have been increasing in southern Europe and in other regions [2, 27]. Symptoms range from fever, sore throat and maculopapular rashes to acute hepatitis [18]. The role of *R. aeschlimannii* infection and MSF disease occurrence in humans deserve more study as only a limited number of cases have been reported so far in which the aetiology of MSF has been intensively investigated and correctly attributed. Socio-ecological drivers, including climate change, livestock farming, bird migratory routes and land use practices, may be contributing to the expanding geographic range of *Hyalomma* ticks, thereby increasing human exposure and infection risk.

In Pianosa island, the eradication of non-autochthonous species since 2014 has lowered the available biomass of vertebrate hosts [5, 28–30]. Consequently, residents and tourists increasingly perceive the seasonal presence of *H. marginatum* ticks as a major nuisance, with local social media reporting an alarming picture of 'giant ticks' overwhelming the island and attacking people. Furthermore, the typical Mediterranean ecosystem of the island is facing increased extreme drought and hot conditions [31], together with the re-colonization of abandoned agricultural land by natural vegetation (in particular Mediterranean scrub) [32]. These features, combined with the continuous influx of ticks carried by migratory birds and the dense population of the autochthonous brown hare, may have sustained and favoured the persistence and high density of *Hyalomma* ticks. Consequently, the abundance of *H. marginatum* coupled with the high prevalence rates of *R. aeschlimanni* could increase the hazard of transmission to residents and tourists visiting Pianosa island. Although Park authorities provide some information about the risks of tick exposure, a dedicated action plan aimed at increasing awareness regarding the presence of ticks should be implemented. Such a programme may consist of information campaigns using social media, trained staff, local tour operators and the National Park website, informing also on the best practices to reduce the risk of infection (i.e. use of repellents, protective clothing, restrictions to walking off cleared paths, daily tick body check for people and pets). Targeted control measures using acaricides would need to be evaluated since their use in protected areas is often controversial, and would be limited to selected areas such as the public beaches, recreational areas, the small village and meeting areas. All of these prevention measures may help to reduce the risk of tick exposure and of *R. aeschlimanni* infection for residents and visitors.

In conclusion, this study highlights the high prevalence of *R. aeschlimanni*, a known and emerging human pathogen, in both *H. marginatum* ticks and vertebrate hosts on Pianosa island. Given the importance of the Tuscan Archipelago National Park for avian migratory routes, together with the heavy pressure exerted through climate change and tourism on its fragile ecosystem, the information provided by this study is crucial for raising awareness of the potential health risk caused by arthropod vectors and arthropod-borne disease agents.

The value of small-scale studies should not be overlooked when considered within a wider context. The unexpected adaptation of *Hyalomma* ticks to the only available vertebrate endangered host due to contingent external factors deserves further attention and long-term monitoring to evaluate variations in *Hyalomma* tick abundance and *R. aeschlimannii* prevalence in relation to a broader set of ecological, environmental and climatic factors. Particular attention should also be devoted to the monitoring of the role of migratory birds in the continuous introduction of infected ticks, especially in view of their potential role to carry other dangerous zoonotic infectious diseases.

## Data Availability

The sequences have been submitted to the European nucleotide archive on 26 August 2025 under a single project with PRJEB96402 accession number.
